# Analysis of Key miRNA/mRNA Functional Axes During Host Dendritic Cell Immune Response to *Mycobacterium tuberculosis* Based on GEO Datasets

**DOI:** 10.3390/genes16070832

**Published:** 2025-07-17

**Authors:** Qian Gao, Shuangshuang Bao, Yaqi Sun, Kaixin Zhou, Yan Lin

**Affiliations:** School of Basic Medical Sciences, Beihua University, Jilin 132000, China; g17829917117@163.com (Q.G.); 18543266515@163.com (S.B.); 19806010157@163.com (Y.S.); 15838849770@163.com (K.Z.)

**Keywords:** *Mycobacterium tuberculosis*, dendritic cell, GEO datasets, regulatory network, miRNA/mRNA axis

## Abstract

Background: Dendritic cells (DCs) play an important role as a bridge between innate and adaptive immunity, and changes in gene expression of DCs during the immune response to *Mycobacterium tuberculosis* (*M.tb*) may affect the development of tuberculosis. Methods: Using systems biology methods, mRNA and miRNA expression profile data of DCs infected with *M.tb* were obtained. A total of 1398 differentially expressed mRNAs and 79 differentially expressed miRNAs were identified, and a corresponding miRNA–mRNA regulatory network was constructed using Cytoscape 3.9.1 software. The functional annotations and pathway classifications of the miRNA–mRNA network were identified using the DAVID tool. Then, the key pathway modules in the miRNA–mRNA network were screened and subjected to PPI network analysis to identify hub nodes. Subsequently the miRNA/mRNA axis was determined, validated by qRT-PCR, and evaluated through ROC curve analysis. Results: The TNF signaling pathway and the Tuberculosis pathway were key pathway modules, with miR-34a-3p/*TNF* and miR-190a-3p/*IL1B* being the greatest correlations with the two pathway modules. qRT-PCR results showed that *IL1B* and miR-190a-3p exhibited significant differences in both the H37Ra and BCG infection groups. The AUC of two factors (*IL1B* and miR-190a-3p) was 0.9561 and 0.9625, respectively, showing high sensitivity and specificity. Conclusions: Consequently, miR-190a-3p/*IL1B* might be a good candidate marker to characterize the immune response of DCs to *M.tb* and a transition signal from innate to adaptive immunity.

## 1. Introduction

Tuberculosis (TB) is one of the major chronic infectious diseases in the world. According to the World Health Organization (WHO), in 2023 [1], *M.tb* infection led to 10.8 million new TB cases globally and an incidence rate of 134/100,000, with China estimating the number of new TB patients at 741,000 and the TB incidence at 52/100,000. There were 1.25 million global deaths due to TB, with TB regaining status as the top single infectious disease cause of death globally, causing almost twice as many deaths as HIV/AIDS [1]. The host immune response to *M.tb* involves a complex innate and adaptive immune response network [2]. Innate immune cells are the first to encounter *M.tb*, and their response dictates the course of infection [3]. DCs are one of the key cell types that mediate innate and adaptive immune responses and play an important role in capturing, processing, and presenting antigens [2]. DCs can also activate the adaptive response and determine its characteristics [3,4].

A number of studies have shown that DCs can enhance their own cellular functions to initiate the immune response against *M.tb* infection [5,6,7]. Immature DCs exist in the lung mucosa [2] and recognize pathogen-related molecular patterns through pattern recognition receptors (PRRs), which is accompanied by phagocytosis and microbial internalization that further leads to a maturation process [8]. Mature DCs are characterized by high expression of MHC class II (MHC II) on their surface and expression of costimulatory molecules such as CD40, CD80, and CD86 [8]. Furthermore, DCs secrete IL12 to prime T cells. *M.tb* can disrupt the maturation of DCs as well as reduce their ability to secrete IL12 and stimulate T cell proliferation [9,10]. When mature DCs migrate to secondary lymphoid organs, they present pathogen-derived antigens to naive T cells, initiate the activation and differentiation of these T cells, and play a key role in determining the types of Th subpopulations generated by infection [11,12,13]. There is an increasing amount of evidence showing that in the process of high-frequency infection of humans and mice by *M.tb*, DCs affect the immune function against TB, both in vitro and in vivo [14,15,16]. It has been reported that the presentation ability of dendritic cells has great potential for the design of synthetic vaccines against tuberculosis [17]. Thus, DCs play a central role in immunity against microbial pathogens by connecting innate and adaptive immune responses [11,18].

The immune function of DCs, mobilized during *M.tb* infection, inevitably causes changes in the expression of some genes. Luis B. Barreiro et al. studied primary DCs from 65 individuals and found changes in the regulatory immune response of DCs infected with *M.tb*, mostly related to genetic factors and gene expression; specifically, the maturation of DCs after *M.tb* infection was accompanied by the strong upregulation of genes involved in immune responses (FDR < 10^−26^) [19]. Transcriptome changes in DCs after *M.tb* infection are expected to reflect the transformation of DCs from antigen-capture cells to strong antigen-presenting cells and T cell activators [20,21]. Similarly, recent studies have shown that a large number of microRNAs (miRNAs) are involved in the response to *M.tb* [22,23,24,25]. miRNAs in PBMCs can be expressed endogenously during the host immune response to *M.tb* and play a role in regulating gene expression at the post-transcriptional level. Additionally, miRNAs are involved in the regulation of the development and function of immune cells and even in the associated proinflammatory or anti-inflammatory effects [26,27,28]. Katherine J. Siddle et al. have used deep sequencing technology to describe the miRNA transcriptional response of DCs during *M.tb* infection over time, and highlighted common miRNA-mediated mechanisms that may be critical in cellular responses to stress [29]. Therefore, by identifying key miRNAs and mRNAs within the expression profiles of DCs infected with *M.tb*, we can identify those that are differentially expressed and combine them to construct an aberrant miRNA–mRNA regulatory network for the study of the DCs’ immune response to *M.tb* infection. It is of great significance to explore the mechanism of host immune response to *M.tb* by understanding the characteristics of the process through which DCs respond to *M.tb*.

## 2. Materials and Methods

### 2.1. Expression Profile Data Collection and Analysis

Using the Gene Ontology (GEO) datasets of the National Center for Biotechnology Information (NCBI), a total of 259 mRNA microarray cases from *M.tb*-infected and uninfected DCs, as well as 36 miRNA RNA-seq data cases from infected and uninfected DCs, were collected. The Limma and edgeR packages of the R language (version 4.2.1) were applied to screen mRNAs and miRNAs differentially expressed between the infected and control groups. Conditions were considered different when *p* < 0.05 and |Log2| ≥ 1 (Fold change ≥ 2 or Fold change ≤ 0.5). Then, the ggplot2 package of the R language was used for a statistical analysis to establish the expression trend of the differentially expressed mRNAs and miRNAs screened.

### 2.2. Establishment of an miRNA–mRNA Regulatory Network Related to DCs Infected with M.tb

According to the results of the analysis of the mRNA and miRNA expression profile data, differentially expressed mRNAs and miRNAs in DCs exhibiting an immune response to *M.tb* were screened. The target genes predicted by differentially expressed miRNAs were inferred from the Targetscan, miRDB, and miRWalk2.0 databases. An intersection analysis was performed using the differentially expressed mRNAs generated from the expression profile data analysis. The miRNA–mRNA regulatory network associated with *M.tb* infection of DCs was established by the Cytoscape software (version 3.9.1). In this regulatory network, the pink V symbols represent miRNAs, and the blue ellipses the predicted target genes, which may show an abnormal relationship in DCs infected with *M.tb*. miRNAs and their target mRNAs were connected by dotted lines from the source to the target. The DIANA TOOLs-mirPath was used to analyze miRNAs and could give further insight into the signaling pathways influenced by the DCs’ immune response to *M.tb*. When compared to control, a *p* value less than 0.5 (*p* < 0.5) denoted statistical significance.

### 2.3. Functional Annotation and Pathway Enrichment Analysis for the miRNA–mRNA Regulatory Network

The Database for Annotation, Visualization, and Integrated Discovery (DAVID) online analysis tool and the Kyoto Encyclopedia of Genes and Genomes (KEGG) database were used to analyze the functional annotation and pathway enrichment of differentially expressed genes in the miRNA–mRNA regulatory network identified in DCs during their immune response to *M.tb*. The analysis results were visualized by the R language ggplot2 package.

### 2.4. PPI Network Analysis of Key Modules

The target genes of the miRNA–mRNA regulatory network abnormally expressed in DCs infected with *M.tb* were analyzed by the Search Tool for the Retrieval of Interacting Genes (STRING, http://string-db.org/, accessed on 1 May 2024) [30] database, and were used to perform a protein–protein interaction (PPI) network analysis. PPIs were displayed in the form of a network by Cytoscape 3.9.1, with nodes of different colors showing the degree of correlation for each gene. The aim was to find key function modules that may play an important role in the immune process of the host DCs during *M.tb* infection, and from which we could discover an important miRNA/mRNA axis that may be a starting point for future research in the field.

### 2.5. Propagation and Infection of M.tb H37Ra, BCG, and DCs

The attenuated strain of *M.tb* H37Ra was purchased from the American Type Culture Collection (ATCC), catalog number ATCC^®^ 25177™. The BCG was obtained from the BeNa Culture Collection (BNCC). Both H37Ra and BCG were grown in Middlebrook 7H9 medium (BD Biosciences, Franklin Lakes, NJ, USA) and cultured at 37 °C for 15~20 days, supplemented with 10% ADC (BD Biosciences), 0.2% glycerin, and 0.05% Tween-80.

Human peripheral blood mononuclear cells (PBMCs) were purchased from Beijing Nuopu Biological Co., Ltd. (Beijing, China). The cells were cultured in RPMI-1640 medium for 2 h, after which the suspended lymphocytes were removed, and the adherent monocytes were retained and collected. These cells were then cultured in T25 culture bottles containing RPMI-1640 medium supplemented with 10% fetal bovine serum (FBS), granulocyte-macrophage colony-stimulating factor (GM-CSF, final concentration of 100 ng/mL), and interleukin-4 (IL-4, final concentration of 100 ng/mL). The culture was maintained in a 37 °C incubator with 5% CO_2_ atmosphere. On the 6th day of cell culture, tumor necrosis factor-alpha (TNF-α, final concentration of 20 ng/mL) was added to the medium, and the cells were further incubated to induce DCs maturation. Mature DCs were harvested on the 8th day.

The DCs derived from PBMCs were inoculated in 6-well plates, infected with H37Ra and BCG under the condition of an MOI of 1, and incubated in a 37 °C, 5% CO_2_ incubator for 24 h.

### 2.6. Laser Confocal Fluorescence Co-Localization Analysis

By observing the co-localization of *M.tb* with lysosomes in DCs, the infection model was established successfully. Fluorescein isothiocyanate (FITC) and LysoTracker Red DND-99 were purchased from Good Laboratory Practice Bioscience (GLPBIO).

First, H37Ra and BCG in the middle stage of exponential growth (OD value reaching 0.7) were collected and prepared into a bacterial suspension using PBS. FITC (final concentration of 50 µg/mL) was added, and the bacterial suspension was cultured in an incubator at 37 °C for 2 h. After centrifuging at 5000 rpm for 10 min, the supernatant was discarded. The bacteria were washed twice with PBS, resuspended in RPMI-1640 medium, and stored at 4 °C away from light until use.

Second, FITC-labeled *M.tb* suspension was added to DCs cultured on coverslips in a six-well plate. After 24 h of incubation in a 37 °C, 5% CO_2_ incubator, the infected cell culture medium was removed, and the cells were washed three times with PBS. LysoTracker Red DND-99 working solution was added to make a final concentration of 50 nM. After incubation at 37 °C for 90 min, the cells were then fixed with glutaraldehyde for 30 min. The whole operation was kept away from light. Then, the fixative was removed, and the coverslips were mounted on slides dripping with 50% glycerol. The co-localization of H37Ra, BCG, and lysosomes was observed using a laser confocal microscope at 20× magnification.

### 2.7. RNA Extraction and Identification of Key miRNA and mRNA by qRT-PCR

Total RNA was extracted using a FastPure Cell Total RNA Isolation Kit (Vazyme, Nanjing, China), and the extracted total RNA was reverse transcribed into cDNA using HiScript II Reverse Transcriptase (Vazyme, Nanjing, China). qRT-PCR analysis was performed using ChamQ Universal SYBR qPCR Master Mix (Vazyme, Nanjing, China), with all experimental procedures strictly following the kit instructions. miRNAs were extracted from the samples using miRcute miRNA isolation kit (Tiangen, Beijing, China) according to the instructions, and the miRNA expression was verified by miRcute Plus miRNA qPCR Kit (SYBR Green) (Tiangen). The relative expression levels of differential miRNAs and mRNAs were calculated using the 2^−∆∆Ct^ method (relative quantification). Sangon Biotech (Shanghai, China) Co., Ltd. designed and synthesized the primers, and the primer sequences are shown in Table 1. The qRT-PCR results were analyzed using GraphPad Prism (version 8.0). The differences between groups were analyzed using a one-sided statistical analysis with a Student’s *t*-test, and *p* < 0.05 was considered significant.

### 2.8. Statistical Analysis of Key miRNA/mRNA Axes

The receiver operating curve (ROC) was used to analyze the expression characteristics of the key miRNA/mRNA axes in DCs responding to the immune response of *M.tb*. The ROC curve was plotted using GraphPad Prism 8.0 software, and the area under the curve (AUC) was calculated to determine the specificity and sensitivity of the abnormal expression axes. A *p*-value less than 0.05 was considered statistically significant.

## 3. Results

### 3.1. Screening Results for Differentially Expressed mRNAs and miRNAs in DCs Infected with M.tb

**Figure 1:**
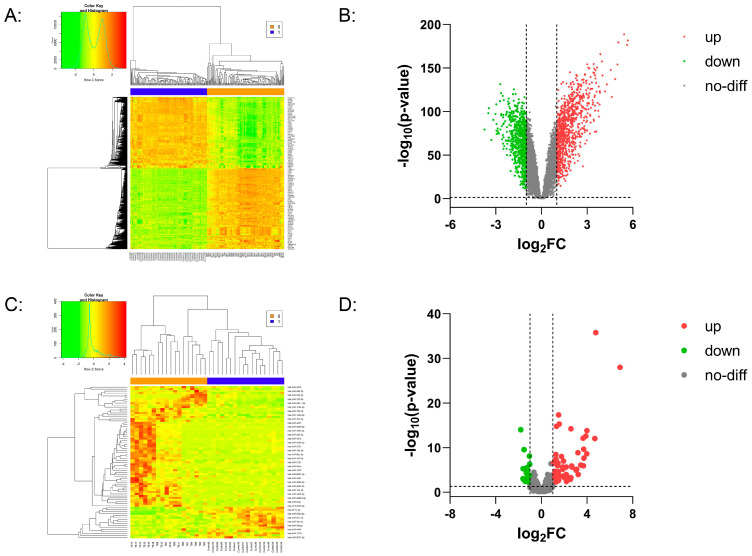
Visualization analysis of differentially expressed mRNAs and miRNAs in DCs infected with *M.tb*. (**A**) Cluster analysis of differentially expressed mRNAs in DCs infected with *M.tb* vs. non-infected samples; (**B**) Volcano plot of differentially expressed mRNAs; (**C**) Cluster analysis of differentially expressed miRNAs in DCs infected with *M.tb* vs. non-infected samples; (**D**) Volcano plot of differentially expressed miRNAs. In the clustering analysis plots; each row represents mRNA or miRNA; each column represents a sample. The “TB” and “Control” columns at the bottom indicate the experimental and control groups, respectively. Red indicates higher expression in the experimental group compared with the control group, while green indicates lower expression.

From GEO datasets, we collected 259 microarray cases, including 130 microarray cases of DCs infected with *M.tb* and 129 microarray cases of non-infected DCs, which were all from the GSE34151 dataset (https://www.ncbi.nlm.nih.gov/geo/query/acc.cgi?acc=GSE34151, accessed on 5 March 2024) (Table S1). According to the experimental design of this dataset, DCs from normal human monocytes in vitro were used as the experimental group after being infected by *M.tb*, while non-infected monocyte-derived DCs served as the control group. By screening the expression profile data, a total of 1398 differentially expressed mRNAs were identified in the experimental group (DCs infected with *M.tb*), among which 739 mRNAs were up-regulated and 659 mRNAs were down-regulated (Table S2). Cluster analysis showed significant differences in mRNA expression trends between the experimental group and the control group (Figure 1A). A volcano plot was generated for the 1398 differentially expressed mRNAs. In the plot, red dots represent up-regulated genes, green dots indicate down-regulated genes, and grey dots represent genes with no significant difference (Figure 1B). Similarly, a total of 36 miRNA RNA-seq data cases were collected from GEO datasets, including 18 cases from *M.tb*-infected DCs and 18 cases from uninfected DCs, which were all from the GSE64142 dataset (https://www.ncbi.nlm.nih.gov/geo/query/acc.cgi?acc=GSE64142, accessed on 7 March 2024.) (Table S3). According to the experimental design of this dataset, normal human monocyte-derived DCs following infection with a panel of *M.tb* in vitro also served as the experimental group, and non-infected monocyte-derived DCs as the control group. Based on sequencing data analysis, 79 differentially expressed miRNAs were identified in the experimental group (DCs infected with *M.tb*), among which 62 were up-regulated and 17 were down-regulated (Table S4, Figure 1D). Cluster analysis showed that the expression trend of miRNAs was also significantly different between the experimental group and the control group (Figure 1C).

### 3.2. Construction and Analysis of Potential miRNA–mRNA Regulatory Network for the Immune Response of DCs to M.tb

**Figure 2:**
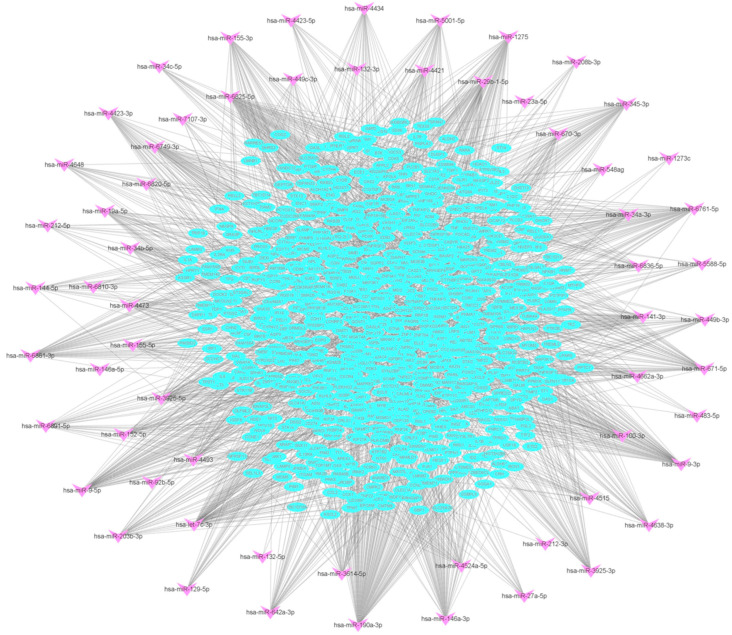
miRNA–mRNA regulatory network in the DCs’ immune response to *M.tb* infection. The miRNA–mRNA regulatory network, which was abnormally expressed in DCs upon *M.tb* infection, consisted of 62 miRNAs and 832 mRNAs. Pink V symbols represent miRNAs, and blue ellipses represent miRNAs. The connecting lines extend from the miRNA to the mRNA.

**Figure 3:**
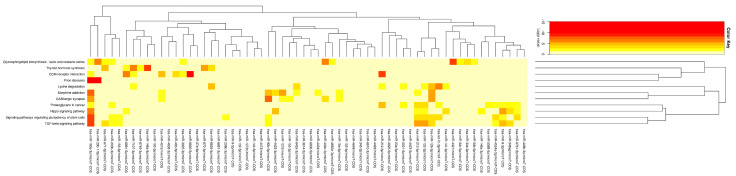
Heatmap of statistically significant correlations between the 62 miRNAs and their mediated pathways based on *p* values (log scale). Red represents a high level of significance.

Using the differentially expressed mRNAs and miRNAs associated with the DCs’ immune response to *M.tb*, in combination with the Targetscan, miRDB, and miRWalk2.0 databases, we performed an intersection analysis and established the potential miRNA–mRNA regulatory network that is abnormally expressed in DCs infected with *M.tb* (Figure 2). The network contained 62 miRNAs and 832 mRNAs (Table S5). To further investigate the role of this network in the DCs’ immune response to *M.tb* infection, we analyzed the miRNA signaling pathways in the network by DIANA Tools-mirPath [31]. This analysis showed that the miRNAs in the miRNA–mRNA regulatory network associated with the DCs’ immune response to *M.tb* infection were enriched in ECM-receptor interactions (eight miRNAs), the TGF-beta signaling pathway (11 miRNAs), and the Hippo signaling pathway (10 miRNAs), among others (Figure 3, Table 2). All of these pathways are related to the course of infection [32,33,34,35].

### 3.3. Functional and Pathway Analysis of Differentially Expressed Genes in miRNA–mRNA Regulatory Networks from DCs Infected with M.tb

**Figure 4:**
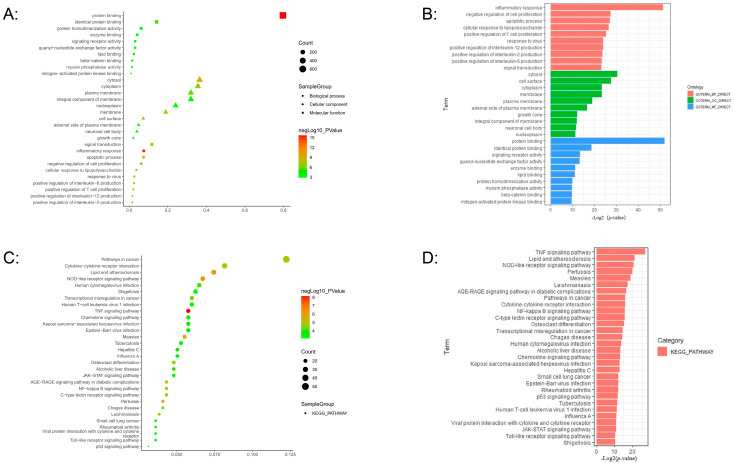
Functional annotation and pathway classification of 832 differentially expressed genes in the miRNA–mRNA regulatory network, with the top 30 genes listed. (**A**,**B**) Gene ontology analysis; (**C**,**D**) KEGG analysis.

The DAVID online analysis tool integrated with the KEGG database was used to analyze 832 differentially expressed genes in the miRNA–mRNA regulatory network related to the DCs’ response to *M.tb*. Functional annotation showed that these genes were closely related to inflammatory responses, apoptotic processes, and immune responses, among others (Figure 4A,B). Subsequent pathway classification showed that the TNF signaling pathway was the most significant, followed by the NOD-like receptor signaling pathway, while the Lipid and atherosclerosis pathways were also prominently present in the classification. Moreover, the Tuberculosis pathway was also significantly enriched in the gene network (Figure 4C,D).

### 3.4. Acquisition of Pivotal Axes in the miRNA–mRNA Regulatory Network by PPI Analysis

**Figure 5:**
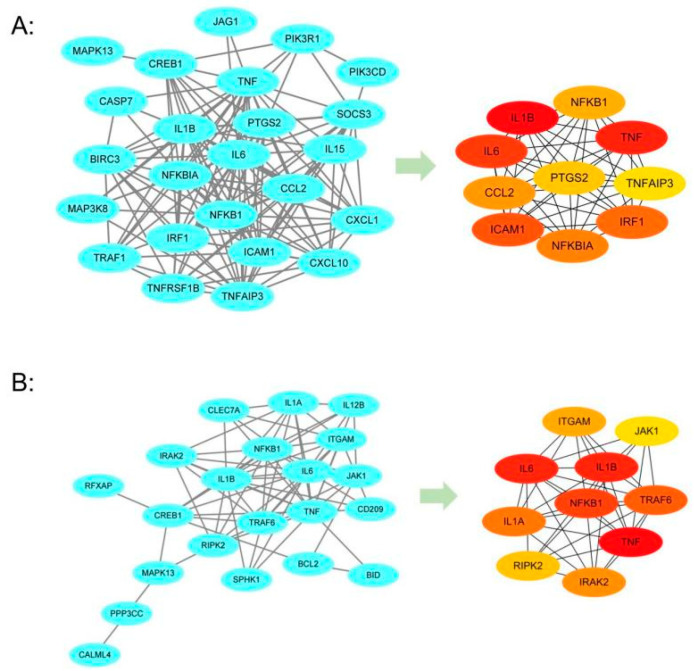
PPI network and module analysis of two pathways. (**A**) The network and modules in the TNF signaling pathway; (**B**) The network and modules in the Tuberculosis pathway; Darker color denotes higher ranking for the top 10 genes.

Based on the results of the pathway classification analysis of the genes belonging to the miRNA–mRNA regulatory network, the most prominent signaling pathway identified was the TNF signaling pathway. At the same time, the Tuberculosis pathway was statistically significant. Therefore, we subsequently focused on the analysis of the important genes enriched in these two pathways, and their related miRNAs. First, for these genes that belonged to the miRNA–mRNA regulatory network and were enriched in the TNF signaling pathway and the Tuberculosis pathway, we analyzed the connections among proteins using STRING (http://string-db.org/). The results were visualized in Cytoscape 3.9.1 and analyzed by the Cytohubba tool to identify hub nodes. In the PPI network of the TNF signaling pathway, we identified 24 mRNAs, which are marked by a blue oval (Figure 5A), suggesting that there may be an important relationship between the proteins encoded. Then, using the Cytohubba tool to identify hub genes calculated by the MCC algorithm, we found an important module composed of the top 10 genes (Figure 5A), ranked as follows: *IL1B*, *TNF*, *IL6*, *ICAM1*, *IRF1*, *NFKBIA*, *CCL2*, *NFKB1*, *PTGS2*, *TNFAIP3* (Table 2). In the PPI network of the Tuberculosis pathway, we found 21 mRNAs marked by the blue oval (Figure 5B), and we also obtained an important module with the following ranking order: *TNF*, *IL6*, *IL1B*, *NFKB1*, *TRAF6*, *IL1A*, *IRAK2*, *ITGAM*, *RIPK2*, *JAK1* (Table 3). We found that the first three important genes were *TNF*, *IL6*, and *IL1B* in both of these two important pathway modules.

### 3.5. Mining and Statistical Analysis of Important miRNA/mRNA Axes

Based on the above analysis, we identified important functional modules in the TNF signaling pathway and Tuberculosis pathway in the miRNA–mRNA regulatory network during the DCs’ immune response to *M.tb*. It was found that the top three genes in the modules from the two pathways were *TNF*, *IL6*, and *IL1B*. According to the information in the miRNA–mRNA regulatory network, hsa-miR-34a-3p, hsa-miR-3925-5p, and hsa-miR-190a-3p were the miRNAs with a predictive target gene regulation relationship with the *TNF*, *IL6*, and *IL1B* genes, respectively. Due to the high encoding number of hsa-miR-3925-5p and the limited research data currently available on it, we focused our analysis on miR-34a-3p/*TNF* and miR-190a-3p/*IL1B*. During the immune response of DCs infected with *M.tb*, two miRNA/mRNA axes, namely miR-34a-3p/*TNF* and miR-190a-3p/*IL1B*, may play important roles through the TNF signaling pathway and the Tuberculosis pathway. qRT-PCR was used to detect the expression levels of *TNF*, *IL1B*, miR-34a-3p, and miR-190a-3p in H37Ra- and BCG-infected and uninfected DC models.

### 3.6. M.tb Successfully Entered the Lysosomes of PBMC-Derived DCs

To further analyze whether the abnormal expression of miR-34a-3p/*TNF* and miR-190a-3p/*IL1B* was related to pathological processes during the DCs’ immune response to *M.tb* infection, we first modeled the DCs infected by *M.tb*. DCs were infected with the attenuated H37Ra strain and BCG, and their co-localization images were captured using a laser confocal microscope.

**Figure 6:**
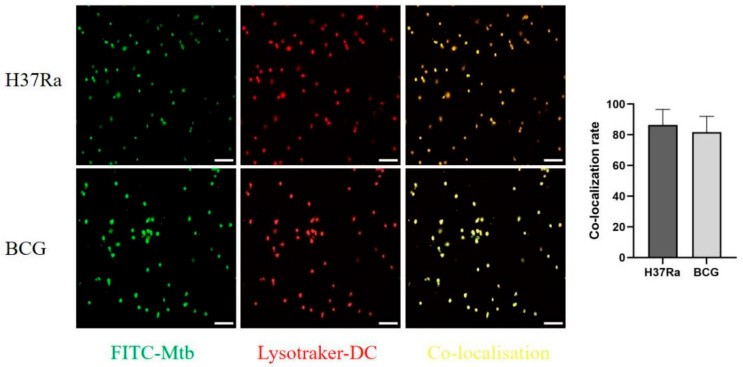
Laser confocal microscopy observations (50 μm) and statistical analysis results for DCs infected with *M.tb*.

The results showed green fluorescence from FITC-labeled *M.tb* (H37Ra and BCG) and red fluorescence from lysosomes of DCs stained with LysoTracker Red DND-99. The overlap of green fluorescence and red fluorescence indicated the successful establishment of the infection model. The yellow fluorescence resulting from the overlap represented the co-localization of *M.tb* (H37Ra and BCG) and DCs. As shown in Figure 6, the results indicate that *M.tb* (H37Ra and BCG) successfully infected DCs.

### 3.7. Validation of Differential miRNA Expression Levels via qRT-PCR

**Figure 7:**
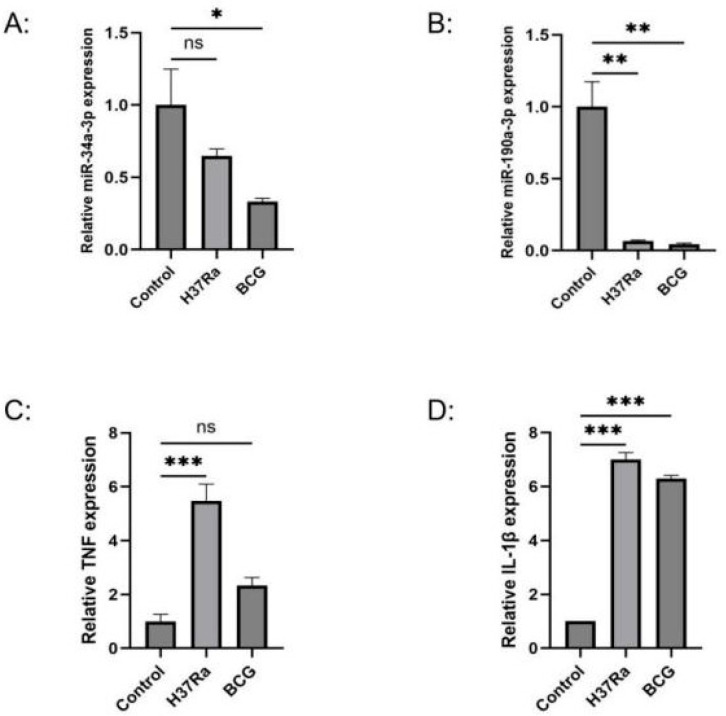
qRT-PCR validation of key miRNA and mRNA expression levels. (**A**) Relative expression level of miR-34a-3p; (**B**) Relative expression level of miR-190a-3p; (**C**) Relative expression level of *TNF*; (**D**) Relative expression level of *IL1B* (* *p* < 0.05, ** *p* < 0.01, *** *p* < 0.001).

miR-34a-3p and miR-190a-3p were verified by qRT-PCR, and the relative expression differences were calculated using the 2^−∆∆Ct^ method with U6 as the internal control. The results showed that miR-34a-3p exhibited no significant difference in the H37Ra attenuated strain infection group but was down-regulated in the BCG infection group, with relative expression values of 0.65 ± 0.09 and 0.33 ± 0.04, respectively (Figure 7A). miR-190a-3p was significantly down-regulated in both infection groups, with relative expression values of 0.07 ± 0.01 in the H37Ra group and 0.04 ± 0.01 in the BCG group (Figure 7B).

### 3.8. Analysis of Relative Expression Levels of TNF and IL1B in the Infection Model

*TNF* and *IL1B*, two key genes identified in the study, were selected for validation of their differential expression levels between the infection and control groups using qRT-PCR. The relative expression differences were calculated using the 2^−∆∆Ct^ method with β-actin as the internal control. The results showed that *TNF* was significantly up-regulated in the H37Ra infection group but showed no significant difference in the BCG infection group, with relative expression values of 5.48 ± 1.08 and 2.33 ± 0.52, respectively (Figure 7C). *IL1B* expression was significantly up-regulated after *M.tb* infection, with relative expression values of 7.01 ± 0.45 in the H37Ra group and 6.3 ± 0.19 in the BCG group (Figure 7D).

### 3.9. ROC Curve Analysis of the Expression of miR-190a-3p and IL1B

**Figure 8:**
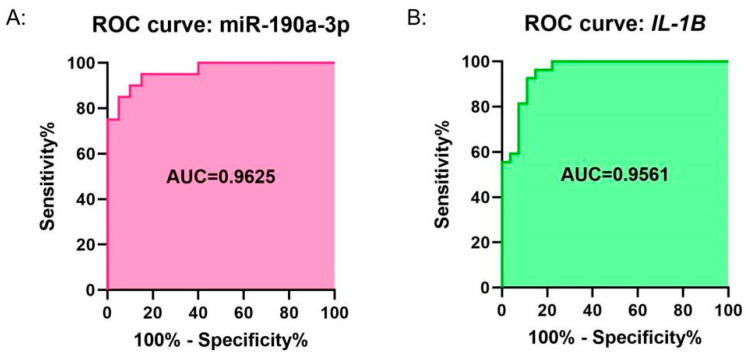
ROC curve analysis of miR-190a-3p/*IL1B* in DCs after *M.tb* infection. (**A**) Efficiency curve of miR-190a-3p in diagnosing tuberculosis. (**B**) Efficiency curve of *IL1B* in diagnosing tuberculosis.

Since miR-190a-3p/*IL1B* showed significant differential expression in the *M.tb* infection groups, we conducted an ROC curve analysis to further explore the correlation between miR-190a-3p/*IL1B* and the clinical characteristics of tuberculosis patients. As shown in Figure 8, the AUC values of miR-190a-3p and *IL1B* were 0.9625 and 0.9561, respectively, both demonstrating high sensitivity and high specificity (Figure 8A,B).

## 4. Discussion

**Figure 9:**
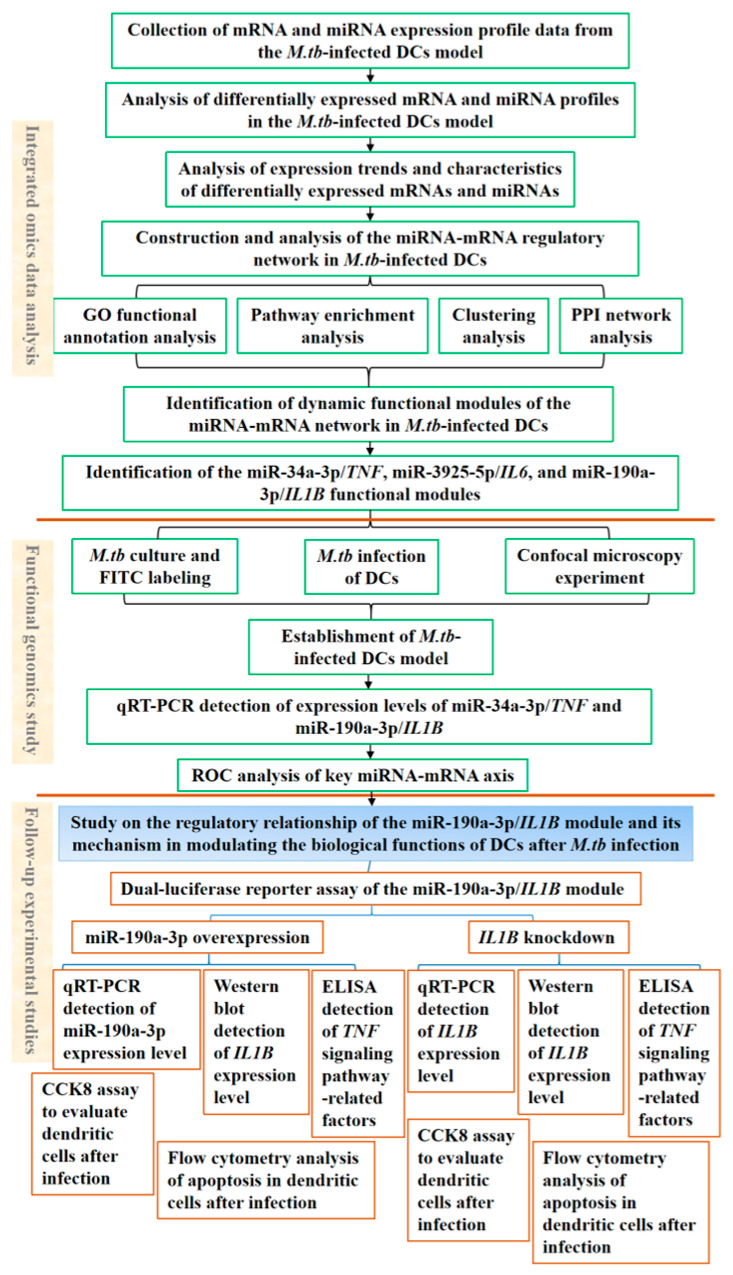
Technical workflow.

In this study, we collected miRNA and mRNA expression profile data of DCs infected with *M.tb*, and screened for differentially expressed miRNAs and mRNAs to establish an miRNA–mRNA regulatory network abnormally expressed during the immune response of DCs to *M.tb*, using the miRNA prediction database (Figure 9). We analyzed the miRNAs in this network using DIANA Tools-mirPath, and found that the aberrantly expressed miRNAs in the network are closely related to ECM receptor interactions, the TGF beta signaling pathway, and the Hippo signaling pathway, which is consistent with recent findings that these pathways play a critical role in regulating the immune system [36,37,38,39]. Subsequently, GO and KEGG analyses were carried out for the target genes in the network. It was found that these abnormally expressed genes were closely related to inflammatory responses, apoptotic processes, and immune responses. At the same time, most of the genes were significantly enriched in the TNF signaling pathway, while enrichment in the Tuberculosis pathway was also statistically significant. Therefore, we conducted PPI network analysis on the TNF signaling pathway genes and Tuberculosis pathway genes, and obtained important pathway gene modules for each pathway. According to this analysis, *TNF*, *IL6*, and *IL1B* were ranked as the top three genes in both pathways. Their abnormal expression may affect the DCs’ immune response to *M.tb* through the TNF signaling pathway, and they may also be related to the Tuberculosis pathway. According to the miRNA–mRNA regulatory network, we extracted miRNAs associated with *TNF*, *IL6*, and *IL1B*, namely hsa-miR-34a-3p, hsa-miR-3925-5p, and has-miR-190a-3p, which constitute the three axes of miR-34a-3p/*TNF*, miR-3925-5p/*IL6*, and miR-190a-3p/*IL1B*. Since hsa-miR-3925-5p has a high coding number and currently available research data on it are limited, we focused on analyzing the potential role of the miR-34a-3p/*TNF* and miR-190a-3p/*IL1B* axes in the immune response process following DCs infection with *M.tb*. qRT-PCR was used to detect the relative expression levels of *TNF*, *IL1B*, miR-34a-3p, and miR-190a-3p in DC models infected and uninfected with H37Ra and BCG. The results showed that miR-34a-3p exhibited no significant difference in the H37Ra attenuated strain infection group but was down-regulated in the BCG infection group. *TNF* was significantly up-regulated in the H37Ra infection group but showed no significant difference in the BCG infection group. miR-190a-3p/*IL1B* exhibited significant differential expression in both infection groups. Compared with the control group, miR-190a-3p was significantly down-regulated in the infection groups, while *IL1B* was significantly up-regulated. To further investigate, ROC curve analysis was performed to evaluate the clinical correlation of miR-190a-3p/*IL1B* with DCs’ immune response to *M.tb*. The results showed that both factors on this axis exhibited high sensitivity and high specificity, suggesting that they might be a marker worth studying to characterize the DCs’ immune response to *M.tb*.

DCs are essential antigen-presenting cells (APCs) that activate T lymphocytes through direct cell–cell interaction and/or cytokine production, and they coordinate innate immunity and adaptive immunity [40,41]. In humans and mice, there are several subtypes of DCs that are characterized by different surface markers and functions. In general, DC subpopulations are derived from bone marrow precursors, which colonize peripheral tissues through the blood or lymphatic system [39,41]. Lung DCs are distributed in the subcutaneous, interstitial, and pleural cavities, and usually exist in the form of immature antigen-presenting cells. Because DCs have the ability to initiate adaptive immune responses, they are considered to be the main targets for regulating immune responses [42]. DCs can sense the presence of *M.tb*. It is precisely because of the inflammatory state of *M.tb* infection that DCs migrating into drainage lymph nodes can be induced to mature. DCs secrete mediators and generate inflammatory signals that drive the activation and differentiation of T lymphocytes necessary for clearing the *M.tb* infection [43]. At the same time, DCs regulate immunity and inflammation and respond to various stimuli, including cytokines [44].

We discovered that the important hub nodes (*TNF* and *IL1B*) were cytokines. The protein encoded by *IL1B* (interleukin 1 beta) is a member of the interleukin 1 cytokine family. This cytokine is an important mediator of the inflammatory response and participates in a variety of cellular activities, including cell proliferation, differentiation, and apoptosis [45,46]. Recent mechanistic studies in mouse models have shown that *IL-1B*-mediated granulocyte inflammation is a potential driver of TB progression [47,48]. *IL-1B* is a typical proinflammatory cytokine that can stimulate local and systemic responses [49]. This cytokine has complex dual roles in chronic infection. The production of *IL-1B* is important for the development of antibiotic-adaptive immunity in the early stages of infection [48,49]. While the production of *IL-1B* is related to the severity of human TB [48], the contradictory activity of *IL-1B* in promoting antibacterial immunity and chronic tissue damage makes the final contribution of this cytokine to the progression of human TB unclear. Although *IL-1B* production by macrophages is essential for protective immunity against the acute phase of TB infection [50,51], during the chronic phase of TB infection, the production of *IL-1B* is suppressed by Th1 immune responses via nitric oxide production to prevent lung damage that could be caused by the prolonged production of *IL-1B* and the associated neutrophil infiltration [47,51,52]. Indeed, several clinical reports showed that *IL-1B* is also involved in TB pathogenesis. There is significantly increased *IL1B* mRNA in the alveolar macrophages and *IL-1B* protein in bronchoalveolar lavage fluid of TB patients compared with healthy controls [53]. Patients with TB pleurisy also had higher *IL-1B* in their serum and pleural fluid than patients with lung cancer [54]. Moreover, a high-*IL1B*-expressing genotype correlated with disease progression and poor treatment outcomes in TB patients [55]. These observations suggest that *IL-1B* production also correlates with the severity of TB. Therefore, it is critical to delineate the mechanisms of *M.tb* infection [55]. And in Viperin deficiency, DCs infected with *M.tb* produced a higher abundance of *IL-1B* [56,57]. *IL-1B* is a key cytokine in the course of both acute and chronic inflammatory responses, making it indispensable for the protection of the host [44]. Based on our miRNA–mRNA network analysis, *IL1B* has a target gene predictive relationship with miR-190a-3p, and the miR-190a-3p signaling pathway in the network is related to ECM-receptor interactions, as revealed by DIANA Tools-mirPath. There have been studies reporting that miR-190a-3p may impact cell viability, cell apoptosis, and inflammatory factor secretion, and promote ECM degradation in LPS-stimulated human nucleus pulposus cells by the connection with lncRNA PART1 [58,59]. Until now, there has been no study about the role of miR-190a-3p in the response of DCs to infection in TB. However, miR-190a-3p/*IL1B* showed the most prominent sensitivity and specificity in our axis analysis, and it may serve as a starting point for further studying the influence of this axis on the immune response of DCs during *M.tb* infection.

Tumor necrosis factor (*TNF*) encodes a multifunctional proinflammatory cytokine that belongs to the TNF superfamily, and it is a critical host resistance factor against TB [60]. *TNF* also plays a critical role in the containment of chronic and latent *M.tb* infection in humans, primates, and mice [61,62,63]. *M.tb* has the ability to activate the maturation of DCs by promoting the expression of surface molecules and the production of TNF-α [64,65]. Importantly, TNF signaling mediates inflammatory responses and contributes to host protection as well as immune-mediated pathology during *M.tb* infection [66]. The miR-34 family has been reported to play an important role in the progression of apoptotic cell death [67]. We are planning future studies to explore how the miR-34a-3p/*TNF* axis, the significance of which is suggested by our current analysis, affects the immune response of DCs to *M.tb* through TNF signaling.

Furthermore, the mechanism of multilevel molecular synergism of related miRNA/mRNA functional axes during the immune response of DCs to *M.tb* will also be a topic for future studies. First, we plan to establish an appropriate experimental model to further analyze the expression trends and binding sites of miR-190a-3p and *IL1B*. We will also analyze the relationship between the dynamic functional axis miR-190a-3p/*IL1B* and the clinicopathological characteristics of TB, and whether this axis plays an important role in the DCs’ immune response to *M.tb* infection by participating in the TNF signaling pathway. We will actively perform the necessary experiments to verify the results of our analysis. Subsequently, the related functional effects of miR-190a-3p and *IL1B* on the immune response of DCs to *M.tb* infection will be analyzed through overexpression and knockdown experiments. At present, the high-throughput data of DC infection models from other infectious diseases in the GEO database are insufficient to support the analysis of differentially expressed genes, and there is no study reporting the expression of these important functional axes in other similar infectious diseases. Therefore, we will consider establishing other models of DCs infected by similar microorganisms in follow-up experiments, and carry out validation studies to improve our research. Using a systems biology approach to construct an miRNA–mRNA regulatory network and its key modules based on PPI network analysis of significant pathways may facilitate the identification of important functional axes during the immune response of DCs to *M.tb*. The results of this study provide a theoretical basis for further study on the molecular mechanism of important regulatory and functional axes during the immune response of DCs against *M.tb*, which can further provide an analytical basis for mechanistic research, diagnosis, and treatment of TB.

## Figures and Tables

**Table 1:** Primers used for qRT-PCR amplification.

Primer	Primer Sequences
hsa-miR-34a-3p	5′-CAATCAGCAAGTATACTGCCCT-3′
hsa-miR-190a-3p	5′-CTATATCAAACATATTCCT-3′
U6 Forward Primer	5′-CTCGCTTCGGCAGCACA-3′
U6 Reverse Primer	5′-AACGCTTCACGAATTTGCGT-3′
*TNF* Forward Primer	5′-CCACGCTCTTCTGCCTGCTG-3′
*TNF* Reverse Primer	5′-GGCTTGTCACTCGGGGTTCG-3′
*IL1β* Forward Primer	5′-GACCTGGACCTCTCTGCCCTCTG-3′
*IL1β* Reverse Primer	5′-GCCTGCCTGAAGCCCTTGC-3′
*β-actin* Forward Primer	5′-CTGGAACGGTGAAGGTGACA-3′
*β-actin* Reverse Primer	5′-AAGGGACTTCCTGTAACAATGCA-3′

**Table 2:** Three pathways related to the miRNAs in the network analyzed by DIANA TOOLs-mirPath.

KEGG Pathway	*p*-Value	miRNAs
ECM–receptor interaction (hsa04512)	1.217072 × 10^−8^	hsa-miR-190a-3p, hsa-miR-212-5p, hsa-miR-483-5p, hsa-miR-3925-3p, hsa-miR-4515, hsa-miR-6820-5p, hsa-miR-6891-5p, hsa-miR-7107-3p.
TGF-beta signaling pathway (hsa04350)	2.800639 × 10^−8^	hsa-miR-9-3p, hsa-miR-92b-5p, hsa-miR-129-5p, hsa-miR-132-3p, hsa-miR-155-5p, hsa-miR-190a-3p, hsa-miR-203b-3p, hsa-miR-212-3p, hsa-miR-548ag, hsa-miR-671-5p, hsa-miR-4524a-5p.
Hippo signaling pathway (hsa04390)	9.265076 × 10^−5^	hsa-miR-9-3p, hsa-miR-132-3p, hsa-miR-141-3p, hsa-miR-146a-3p, hsa-miR-190a-3p, hsa-miR-212-3p, hsa-miR-548ag, hsa-miR-4423-3p, hsa-miR-6761-5p, hsa-miR-6891-5p.

**Table 3:** Ranking of key nodes for both modules.

Module in TNF Signaling Pathway		Module in Tuberculosis Pathway	
Rank	Node	Rank	Node
1	*IL1B*	1	*TNF*
2	*TNF*	2	*IL6*
3	*IL6*	3	*IL1B*
4	*ICAM1*	4	*NFKB1*
5	*IRF1*	5	*TRAF6*
6	*NFKBIA*	6	*IL1A*
7	*CCL2*	7	*IRAK2*
8	*NFKB1*	8	*ITGAM*
9	*PTGS2*	9	*RIPK2*
10	*TNFAIP3*	10	*JAK1*

## Data Availability

All data generated or analyzed during this study are included in this published article and its Supplementary Information Files.

## Supplementary Materials

The following supporting information can be downloaded at: https://www.mdpi.com/article/10.3390/genes16070832/s1, Table S1. Data collection statistics of mRNA expression profile; Table S2. Information about differentially expressed mRNAs in DCs infected with *M.tb*; Table S3. Data collection statistics of miRNA expression profile; Table S4. Information about differentially expressed miRNAs in DCs infected with *M.tb*; Table S5. miRNA–mRNA regulatory network information regarding the immune response of DCs to *M.tb*.
