# Supplementary material for: Analysis of Key miRNA/mRNA Functional Axes During Host Dendritic Cell Immune Response to Mycobacterium tuberculosis Based on GEO Datasets
Source: Genes (Basel). 2025 Jul 17;16(7):832. doi: 10.3390/genes16070832 (PMC12294207; doi:10.3390/genes16070832)
Supplement: Supplementary file 1 [file genes-16-00832-s001.zip › Table S1.pdf]

Table S1. Data collection statistics of mRNA expression profile.

	***M.tb*-infected**	**Non-infected**
**GSE34151**	GSM842925,GSM842927,GSM842929,<br>GSM842931,GSM842933,GSM842935,<br>GSM842937,GSM842939,GSM842941,<br>GSM842943,GSM842945,GSM842947,<br>GSM842949,GSM842951,GSM842953,<br>GSM842955,GSM842957,GSM842959,<br>GSM842961,GSM842963,GSM842965,<br>GSM842967,GSM842969,GSM842971,<br>GSM842972,GSM842974,GSM842976,<br>GSM842978,GSM842980,GSM842982,<br>GSM842984,GSM842986,GSM842988,<br>GSM842990,GSM842992,GSM842994,<br>GSM842996,GSM842998,GSM843000,<br>GSM843002,GSM843004,GSM843006,<br>GSM843008,GSM843010,GSM843012,<br>GSM843014,GSM843016,GSM843018,<br>GSM843020,GSM843022,GSM843024,<br>GSM843026,GSM843028,GSM843030,<br>GSM843032,GSM843034,GSM843036,<br>GSM843038,GSM843040,GSM843042,<br>GSM843044,GSM843046,GSM843048,<br>GSM843050,GSM843052,GSM843054,<br>GSM843056,GSM843058,GSM843060,<br>GSM843062,GSM843064,GSM843066,<br>GSM843068,GSM843070,GSM843072,<br>GSM843074,GSM843076,GSM843078,<br>GSM843080,GSM843082,GSM843084,<br>GSM843086,GSM843088,GSM843090,<br>GSM843092,GSM843094,GSM843096,<br>GSM843098,GSM843100,GSM843102,<br>GSM843104,GSM843106,GSM843108,<br>GSM843110,GSM843112,GSM843114,<br>GSM843116,GSM843118,GSM843120,<br>GSM843122,GSM843124,GSM843126,<br>GSM843128,GSM843130,GSM843132,<br>GSM843134,GSM843136,GSM843138,<br>GSM843140,GSM843142,GSM843144,<br>GSM843146,GSM843148,GSM843150,<br>GSM843152,GSM843154,GSM843156,<br>GSM843158,GSM843160,GSM843162,<br>GSM843164,GSM843166,GSM843168,<br>GSM843170,GSM843172,GSM843174,<br>GSM843176,GSM843178,GSM843180<br>GSM843182.	GSM842924,GSM842926,GSM842928,<br>GSM842930,GSM842932,GSM842934,<br>GSM842936,GSM842938,GSM842940,<br>GSM842942,GSM842944,GSM842946,<br>GSM842948,GSM842950,GSM842952,<br>GSM842954,GSM842956,GSM842958,<br>GSM842960,GSM842962,GSM842964,<br>GSM842966,GSM842968,GSM842970,<br>GSM842973,GSM842975,GSM842977,<br>GSM842979,GSM842981,GSM842983,<br>GSM842985,GSM842987,GSM842989,<br>GSM842991,GSM842993,GSM842995,<br>GSM842997,GSM842999,GSM843001,<br>GSM843003,GSM843005,GSM843007,<br>GSM843009,GSM843011,GSM843013,<br>GSM843015,GSM843017,GSM843019,<br>GSM843021,GSM843023,GSM843025,<br>GSM843027,GSM843029,GSM843031,<br>GSM843033,GSM843035,GSM843037,<br>GSM843039,GSM843041,GSM843043,<br>GSM843045,GSM843047,GSM843049,<br>GSM843051,GSM843053,GSM843055,<br>GSM843057,GSM843059,GSM843061,<br>GSM843063,GSM843065,GSM843067,<br>GSM843069,GSM843071,GSM843073,<br>GSM843075,GSM843077,GSM843079,<br>GSM843081,GSM843083,GSM843085,<br>GSM843087,GSM843089,GSM843091,<br>GSM843093,GSM843095,GSM843097,<br>GSM843099,GSM843101,GSM843103,<br>GSM843105,GSM843107,GSM843109,<br>GSM843111,GSM843113,GSM843115,<br>GSM843117,GSM843119,GSM843121,<br>GSM843123,GSM843125,GSM843127,<br>GSM843129,GSM843131,GSM843133,<br>GSM843135,GSM843137,GSM843139,<br>GSM843141,GSM843143,GSM843145,<br>GSM843147,GSM843149,GSM843151,<br>GSM843153,GSM843155,GSM843157,<br>GSM843159,GSM843161,GSM843163,<br>GSM843165,GSM843167,GSM843169,<br>GSM843171,GSM843173,GSM843175,<br>GSM843177,GSM843179,GSM843181.
