# Supplementary material for: Analysis of Key miRNA/mRNA Functional Axes During Host Dendritic Cell Immune Response to Mycobacterium tuberculosis Based on GEO Datasets
Source: Genes (Basel). 2025 Jul 17;16(7):832. doi: 10.3390/genes16070832 (PMC12294207; doi:10.3390/genes16070832)
Supplement: Supplementary file 1 [file genes-16-00832-s001.zip › Table S3.pdf]

Table S3. Data collection statistics of miRNA expression profile.

	***M.tb*-infected**	**Non-infected**
**GSE64142**	GSM1565177,GSM1565178, GSM1565179,GSM1565180, GSM1565181,GSM1565182, GSM1565183,GSM1565184, GSM1565185,GSM1565186, GSM1565187,GSM1565188, GSM1565189,GSM1565190, GSM1565191,GSM1565192, GSM1565193, GSM1565194.	GSM1565143,GSM1565144, GSM1565145,GSM1565146, GSM1565147,GSM1565148, GSM1565149,GSM1565150, GSM1565151,GSM1565152, GSM1565153,GSM1565154, GSM1565155,GSM1565156, GSM1565157,GSM1565158, GSM1565159, GSM1565160.
